# Anti-Fibrotic Effect of Natural Toxin Bee Venom on Animal Model of Unilateral Ureteral Obstruction 

**DOI:** 10.3390/toxins7061917

**Published:** 2015-05-29

**Authors:** Hyun Jin An, Kyung Hyun Kim, Woo Ram Lee, Jung Yeon Kim, Sun Jae Lee, Sok Cheon Pak, Sang Mi Han, Kwan Kyu Park

**Affiliations:** 1 Department of Pathology, College of Medicine, Catholic University of Daegu, 3056-6, Daemyung-4-Dong, Nam-gu, Daegu 705-718, Korea; E-Mails: ahj119@cu.ac.kr (H.J.A.); khkim1@cu.ac.kr (K.H.K.); wooramee@cu.ac.kr (W.R.L.); kjy1118@cu.ac.kr (J.Y.K.); patho.dr.lee@gmail.com (S.J.L.); 2 School of Biomedical Sciences, Charles Sturt University, Panorama Avenue, Bathurst, NSW 2795, Australia; E-Mail: spak@csu.edu.au; 3 Department of Agricultural Biology, National Academy of Agricultural Science, RDA, 300, Nongsaengmyeong-ro, Wansan-gu, Jeonju-si, Jeollabuk-do 560-500, Korea; E-Mail: sangmih@korea.kr

**Keywords:** bee venom, renal fibrosis, inflammation, UUO

## Abstract

Progressive renal fibrosis is the final common pathway for all kidney diseases leading to chronic renal failure. Bee venom (BV) has been widely used as a traditional medicine for various diseases. However, the precise mechanism of BV in ameliorating the renal fibrosis is not fully understood. To investigate the therapeutic effects of BV against unilateral ureteral obstruction (UUO)-induced renal fibrosis, BV was given intraperitoneally after ureteral ligation. At seven days after UUO surgery, the kidney tissues were collected for protein analysis and histologic examination. Histological observation revealed that UUO induced a considerable increase in the number of infiltrated inflammatory cells. However, BV treatment markedly reduced these reactions compared with untreated UUO mice. The expression levels of TNF-α and IL-1β were significantly reduced in BV treated mice compared with UUO mice. In addition, treatment with BV significantly inhibited TGF-β1 and fibronectin expression in UUO mice. Moreover, the expression of α-SMA was markedly withdrawn after treatment with BV. These findings suggest that BV attenuates renal fibrosis and reduces inflammatory responses by suppression of multiple growth factor-mediated pro-fibrotic genes. In conclusion, BV may be a useful therapeutic agent for the prevention of fibrosis that characterizes progression of chronic kidney disease.

## 1. Introduction

Chronic kidney disease involves renal inflammation, interstitial fibrosis, and tubular atrophy [1]. Progressive renal fibrosis is the final common pathway for all kidney diseases leading to chronic renal failure [2]. Histologically, renal fibrosis is characterized by interstitial infiltration of mononuclear cells, accumulation of myofibroblasts, proliferation of interstitial fibroblasts, accumulation of extracellular matrix (ECM) proteins, and tubular atrophy [3]. Inflammation is involved in the initiation and maintenance of renal damage, and a decreased inflammatory response results in the loss of renal fibrosis [4]. The classic view on the connection between inflammation and fibrosis is that they are mediated in a paracrine fashion; in which inflammatory cells secrete pro-fibrotic cytokines that act on resident fibroblasts and tubular cells to promote fibrogenesis [5]. The development and progression of renal fibrosis primarily involves differentiation of renal fibroblasts into myofibroblasts and infiltration of inflammatory cells, including dendritic cells, lymphocytes, macrophages, and mast cells [6]. These cells are regulated by numerous cytokines and growth factors, such as transforming growth factor-β1 (TGF-β1), tumor necrosis factor-α (TNF-α), interleukin-1β (IL-1β), IL-6, fibroblast growth factor (FGF), and platelet-derived growth factor (PDGF). Thus, a therapeutic intervention that blocks the activation of these cytokine and growth factor receptors could improve antifibrotic effects to slow progression of renal fibrosis [7]. 

Because a large variety of pathophysiologically distinct diseases converge finally into renal fibrosis, this makes it a unique target for treatment. Unfortunately, there are no effective therapies in most other types of organ fibrosis [8]. Thus, more systematic and safer agents are required. Purified bee venom (BV) is a mixture of natural toxins produced by honeybees (*Apis mellifera*), and has been widely used as a traditional medicine for various diseases, including arthritis, rheumatism, pain, cancerous tumors, and skin diseases [9,10]. However, the anti-fibrotic effects of BV on renal fibrosis have not been reported.

Therefore, this study investigated the anti-fibrotic effect of BV on the expression of pro-inflammatory cytokines and on the activation of growth factors related with the development of progressive renal fibrosis in an animal model of unilateral ureteral obstruction (UUO). 

## 2. Results and Discussion

### 2.1. Histological Examination of the UUO Mice with or without Treatment with Bee Venom

**Figure 1:**
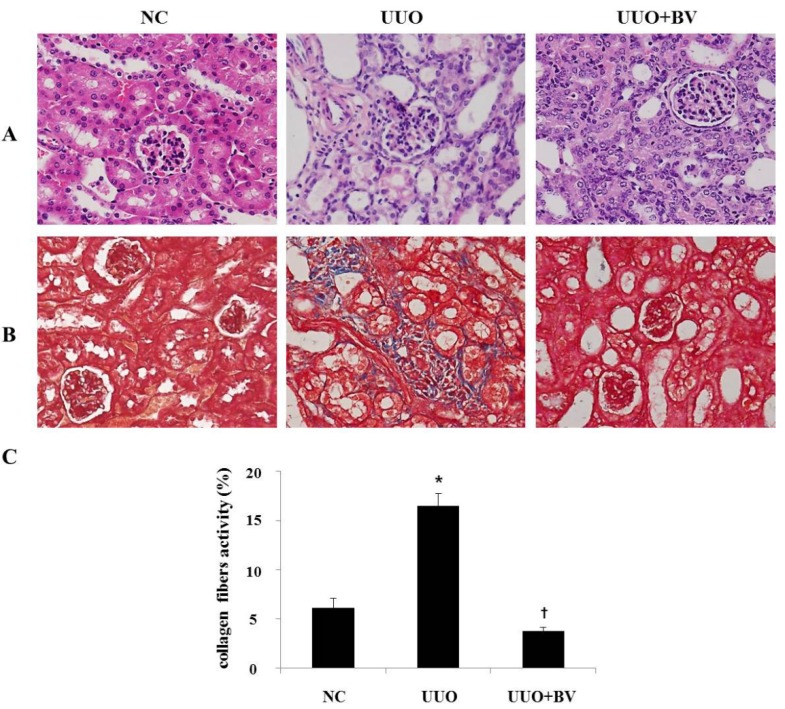
BV inhibits renal fibrosis in obstructed kidney. (**A**) Histological sections of mouse kidney stained with H&E at seven days after UUO surgery. (**B**) Kidney sections are stained with Masson’s trichrome, which accentuates interstitial fibrosis by staining collagen blue. (**C**) Masson’s trichrome staining was used to evaluate the extent of renal fibrosis which was subsequently quantified. NC, normal control; UUO, kidney injury induced by UUO; UUO+BV, UUO treated with 0.01 mg/kg of BV. Representative images from each study group. Magnification 400×. Results are expressed as means ± SE of three independent determinations. * *p* < 0.05 *vs.* NC group. **†** *p* < 0.05 *vs.* UUO group.

The morphological changes in the kidney tissue caused by UUO were visualized in sections stained by hematoxylin and eosin (Figure 1A). Tubular dilatation with flattening of epithelial cells was visualized in UUO kidneys. However, BV treatment significantly reduced these changes when compared to the UUO group. BV attenuated renal histologic damage in UUO mice. The extent of collagen deposition was viewed using Masson’s trichrome staining of renal tissue (Figure 1B) and renal fibrosis was calculated using a well described semiquantitative score derived from the percentage of the positive staining per grid field (Figure 1C). Seven days after UUO surgery, the UUO group demonstrated significant interstitial fibrosis compared with the NC group. However, there was a significant reduction in the number of collagen fibers in the BV treated mice. 

### 2.2. Bee Venom Suppresses Pro-Inflammatory Cytokines in the Kidneys of UUO

**Figure 2:**
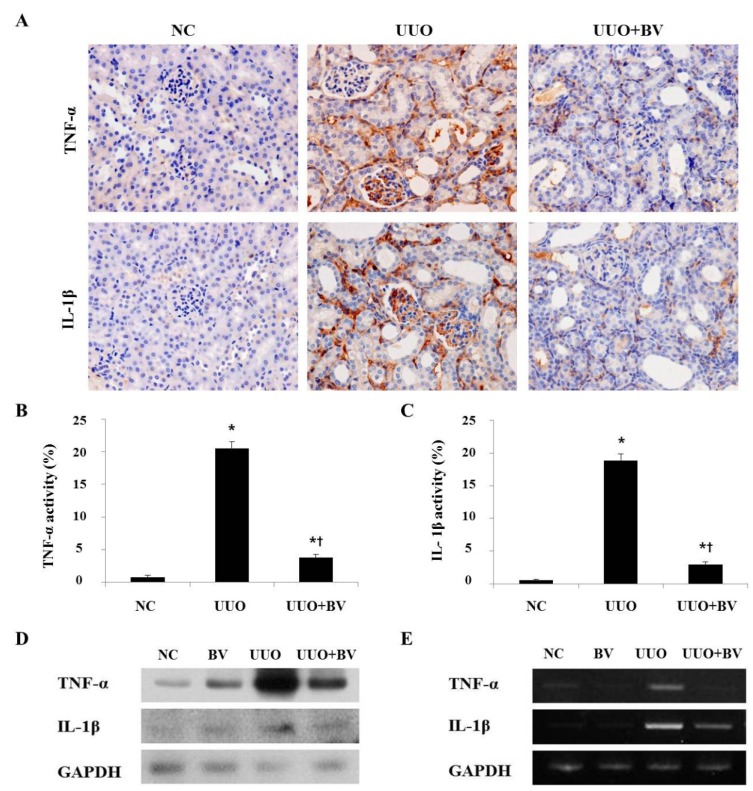
BV attenuates the expression of pro-inflammatory cytokine in obstructed kidneys. (**A**) Representative macrographs show immunohistochemical staining for TNF-α and IL-1β in the kidneys at seven days after UUO surgery. (**B**,**C**) Immunohistochemical staining was used to evaluate the extent of pro-inflammatory cytokines, which was subsequently quantified. (**D**) Western blot analysis shows that BV suppresses the protein expression of TNF-α and IL-1β in UUO kidneys. (**E**) RT-PCR results show that BV suppresses the mRNA expression of TNF-α and IL-1β in UUO kidneys. GAPDH levels were analyzed as an internal control. NC, normal control; UUO, kidney injury induced by UUO; UUO+BV, UUO treated with 0.01 mg/kg of BV. Representative images from each study group. Magnification 400×. Results are expressed as means ± SE of three independent determinations. * *p* < 0.05 *vs.* NC group. **†** *p* < 0.05 *vs.* UUO group.

During UUO, obstruction of the ureter is followed by inflammatory cell infiltration, and by secretion of pro-inflammatory cytokines including TNF-α and IL-1β [11]. To investigate the inflammatory changes in UUO, the expression of TNF-α and IL-1β was determined by immunohistochemical staining, Western blotting and RT-PCR. Immunohistochemistry results showed that UUO kidneys had a marked increase in TNF-α and IL-1β positive cells compared with NC kidneys (Figure 2A). Western blotting and RT-PCR results also demonstrated that the expression of TNF-α and IL-1β was increased in the UUO group (Figure 2D,E). However, the BV treatment group showed significantly reduced expression of pro-inflammatory cytokines compared with the UUO group. There were no obvious expression changes in the kidney of both the NC and BV alone-treated group (Figure not shown). These observations indicate that BV effectively inhibits the expression levels of pro-inflammatory cytokines in UUO mice.

**Figure 3:**
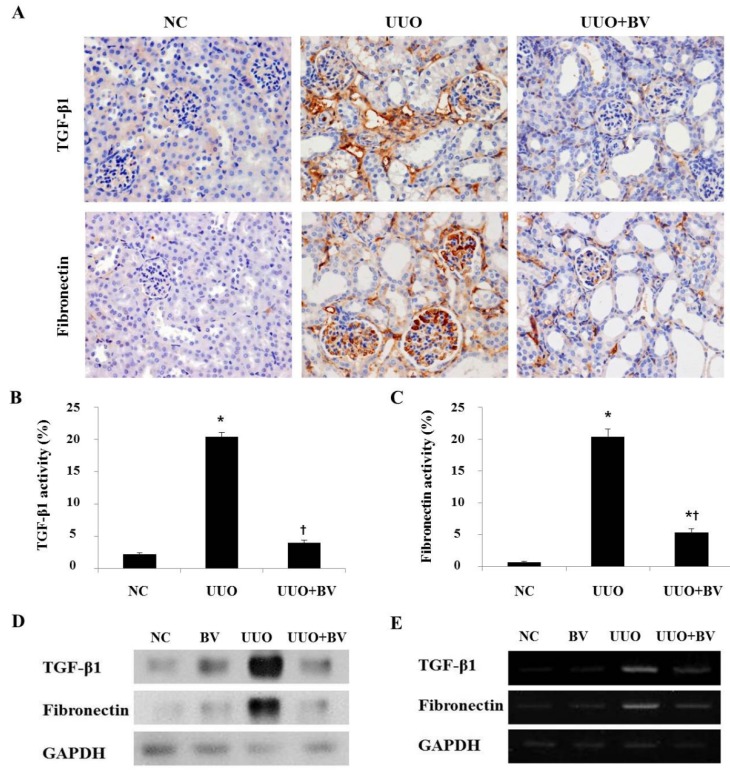
BV attenuates the expression of TGF-β1 and fibronectin in obstructed kidneys. (**A**) Immunohistochemical staining for TGF-β1 and fibronectin in the kidneys at seven days after UUO surgery. (**B**,**C**) Immunohistochemical staining was used to evaluate the extent of fibrotic genes, which was subsequently quantified. (**D**) Western blot analysis shows that BV suppresses the protein expression of TGF-β1 and fibronectin in UUO kidneys. (**E**) RT-PCR results show that BV suppresses the mRNA expression of TGF-β1 and fibronectin in UUO kidneys. GAPDH levels were analyzed as an internal control. Representative images from each study group. Magnification 400×. Results are expressed as means ± SE of three independent determinations. * *p* < 0.05 *vs.* NC group. **†** *p* < 0.05 *vs.* UUO group.

### 2.3. Bee Venom Inhibits the Fibrotic Gene Expression in an Animal Model of UUO

UUO initially produces inflammation, which gradually progresses to fibrosis in the kidney with increased expression of cytokines, such as TGF-β1 [12]. During the development of fibrosis, TGF-β1 expression is upregulated and is known to promote fibrosis under a variety of circumstances, including ECM remodeling [13]. The ECM protein fibronectin is focally deposited in renal fibrosis, where it contributes to inflammatory signaling [14]. As shown in Figure 3A, TGF-β1 and fibronectin positive cells are limited to the tubular basement membranes of the non-obstructed kidney, whereas large amounts of those cells are present in the interstitial space of the obstructed kidney. The cells positive for TGF-β1 and fibronectin were increased in UUO mice, but they were decreased by BV treatment. These observations were confirmed through Western blotting and RT-PCR. The expression of TGF-β1 and fibronectin was increased in UUO, however this increase was abolished by BV treatment (Figure 3D,E). These results suggest that BV effectively blocks fibrotic changes and suppresses the accumulation of ECM in obstructive kidneys.

### 2.4. UUO-Induced Renal Myofibroblast Activation is Suppressed in Bee Venom Treated Mice

**Figure 4:**
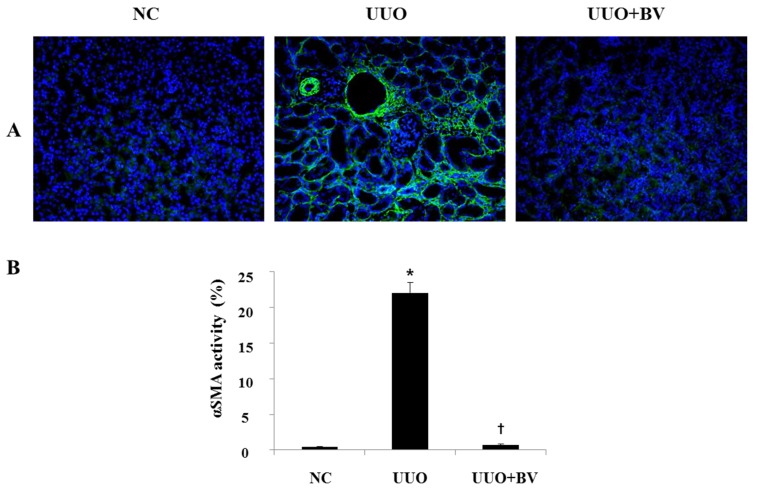
BV abolishes the expression of α-SMA in obstructed kidneys. (**A**) Immunofluorescence staining shows that BV treatment reduces α-SMA positive cells in the kidneys at seven days after UUO surgery. Visible green color indicates α-SMA. Representative images from each study group. (**B**) Immunofluorescence staining was used to evaluate the extent of α-SMA, which was subsequently quantified. Magnification 200×. Results are expressed as means ± SE of three independent determinations. * *p* < 0.05 *vs.* NC group. **†** *p* < 0.05 *vs.* UUO group.

To investigate the ability of BV to suppress myofibroblast activation, this study examined the expression of α-SMA, a representative marker of activated myofibroblasts, by immunofluorescence staining. In normal kidneys, α-SMA positive cells are found only in the blood vessel wall. However, α-SMA positive cells are scattered in the interstitial space of obstructed kidneys, whereas this population of cells was reduced significantly by BV treatment (Figure 4). This data shows clearly that BV plays a critical role in the inactivation of renal fibroblasts after obstructive injury. 

### 2.5. Discussion

Natural toxin BV contains a variety of peptides, including adolapin, apamin, melittin, and mast cell degranulating peptide along with enzymes, biological amines, and other nonpeptide components [10]. In our previous study, we demonstrated that BV can reduce hepatic fibrosis via anti-fibrogenic mechanism [15]. Our another study has reported anti-inflammatory effects of BV against *Propionibacterium* acnes-induced inflammatory skin disease in an animal model [16]. However, the effects of BV during renal fibrosis have not been reported. Thus, this study examined the therapeutic effects of BV on the progression of renal fibrosis using the UUO model. 

Recent reports have shown that obstruction-mediated renal injuries were involved in the mechanisms of inflammatory cytokines, chemokines, and fibrosis-related gene expressions [1,17,18]. TNF-α and IL-1β as key pro-inflammatory cytokines are considered to play important roles in renal fibrosis, and are produced by several types of inflammatory cells [19]. Renal fibrosis is indicated by increasing TGF-β1 activity and collagen deposition, which is stimulated by TNF-α [20,21]. IL-1β is secreted by macrophages in the fibrotic lesions of the kidney [22]. On the basis of this information, this study investigated whether BV could have an effect on these pro-inflammatory cytokines in renal fibrosis. In UUO mice, the numbers of positive cells for TNF-α and IL-1β were increased, but they were decreased by BV treatment. These results demonstrate that BV is an effective blocker of inflammatory cytokine expression. 

TGF-β1 is widely recognized as a strong inducer of fibrosis in renal structures during UUO [23]. During renal fibrosis, TGF-β1 directly regulates the expression of ECM proteins, activates resident fibroblasts and myofibroblasts, and down regulates ECM degradation [13]. Inflammatory reaction in the obstructed kidney stimulates the expression of TGF-β1 [24]. In the present study, the kidneys that received UUO surgery showed increased expression of TGF-β1 and production of fibronectin, a major ECM protein, compared with normal kidneys as demonstrated by immunohistochemistry, Western blot and RT-PCR analyses. However, this increase was abolished by BV treatment in UUO kidneys.

As a consequence of interstitial inflammation, interstitial myofibroblasts were increased and resident interstitial fibroblasts were activated. Interstitial myofibroblasts are the major source of tubulointerstitial ECM and are the best prognostic indicators of disease progression in both human and animal glomerulonephritis [25,26,27]. To investigate the ability of BV to suppress myofibroblast activation *in vivo*, this study examined the effect of BV on the expression of α-SMA, a hallmark of myofibroblasts, in UUO mice. The expression of α-SMA was increased in UUO mice, while this effect was significantly decreased with BV treatment. These results suggest that renal fibrosis is a complex result of various factors and the present study demonstrated that BV can effectively prevent renal fibrosis. 

In summary, these findings suggest that BV attenuates renal fibrosis and reduces inflammatory responses by suppression of multiple growth factor-mediated pro-fibrotic genes. Therefore, BV may be a useful therapeutic agent for the prevention of fibrosis that characterizes progression of chronic kidney disease. 

## 3. Experimental Section

### 3.1. Collection of Bee Venom

Colonies of natural honeybees (*Apis mellifera* L.) used in this study were maintained at the National Academy of Agricultural Science, Korea. BV was collected by the collecting device (Chung Jin Biotech Co., Ltd., Ansan, Korea) in a sterile manner under strict laboratory conditions. In brief, the BV collector was placed on the hive, and the bees were given enough electric shocks to cause them to sting a glass plate, from which dried bee venom was later scraped off. The collected venom diluted in cold sterile water and then centrifuged at 10,000 g for 5 min at 4 °C to discard residues from the supernatant. BV was lyophilized by freeze dryer and refrigerated at 4 °C for later use. BV used in the experiment was confirmed with size exclusion gel chromatography (AKTA Explorer, GE Healthcare, Pittsburgh, PA, USA) by dissolving in 0.02 M phosphate buffer with 0.25M NaCl adjusted to pH 7.2 using a Superdex Peptide column (Amersham Biosciences, GE Healthcare, Pittsburgh, PA, USA).

### 3.2. Animal Model

The animal model was established using male Balb/c mice (20–25 g) that were individually housed in polycarbonate cages and maintained under constant temperature (22 ± 2 °C) and humidity (55%). Mice had free access to food, water and were subjected to an artificial light-dark cycle of 12:12 hours. All surgical and experimental procedures used in current study were approved by the IRB committee at Catholic University of Daegu Medical Center (protocol number 2013-1125-CU-AEC-16-Y). In UUO operation, the abdominal cavity was exposed by a midline incision, and the left ureter was isolated and ligated with 5-0 silk at two points. Balb/c mice were randomly divided into three groups: (1) non-treated mice (Normal Control, NC); (2) UUO mice (UUO); and (3) UUO mice were injected with BV (UUO+BV) (*n* = 6, each group). Intraperitoneal injection of BV at a concentration of 0.01 mg/kg was given immediately after ureteral ligation. Then, BV was given intraperitoneal injection 2 days after UUO operation. The kidneys were collected for mRNA and protein analysis including histologic examination at day 7 post UUO surgery. 

### 3.3. Western Blot Analysis

Tissues were lysed in a lysis buffer (50 mM Tris pH 8.0, 150 mM NaCl, 5 mM EDTA, 0.5% NP-40, 100 mM PMSF, 1 M DTT, 10 mg/mL leupeptin and aprotinin; all from Sigma-Aldrich, St. Louis, MO, USA). After incubation for 30 min on ice, samples were centrifuged at 8000 *g* for 30 min at 4 °C. Then, supernatant was collected. The protein concentration was determined with the Bradford assay (Bio-Rad Laboratories, Hercules, CA, USA). Total protein (10–50 μg) was separated on 8% to 12% SDS-polyacrylamide gels and transferred to PVDF membrane (Millipore Corporation, Bedford, MA, USA) using standard SDS-PAGE gel electrophoresis procedure. Membranes were blocked in 5% skim milk in TBS-T (10 mM Tris, 150 mM NaCl and 0.1% Tween-20) for 2 h at room temperature. Then, membrane was probed with primary antibody for 4 hours and a horseradish peroxidase (HRPO)-conjugated secondary antibody (anti-mouse, anti-rabbit and anti-goat) was used for detection. Signals were detected using an enhanced chemiluminescence detection system (Amersham, Piscataway, NJ, USA). Primary antibodies used in this study were the following: anti-TNF-α, anti-fibronectin, and anti-α-smooth muscle actin (α-SMA, Abcam, MA, USA), anti-TGF-β1 (R&D Systems, Minneapolis, MN, USA), and anti-IL-1β, anti-collagen type I, and anti-glyceraldehyde-3-phosphate-dehydrogenase (GAPDH) from Santa Cruz (Dallas, TX, USA). All primary antibodies were diluted at 1:1000. Signal intensity was quantified by image analyzer (Las 3000, Fuji, Japan).

### 3.4. Histological Analysis

All tissue specimens were fixed in 10% formalin for at least 24 h at room temperature. After fixation, perpendicular sections to the anterior–posterior axis of the kidney were dehydrated in graded ethanol, cleared in xylene, and embedded in paraffin. Thin sections (3 μm) were mounted on glass slides, dewaxed, rehydrated to distilled water, and stained with hematoxylin and eosin (H & E). As part of the histological evaluation, all slides were examined by a pathologist, without knowledge of the previous treatment, under a light microscope.

### 3.5. Immunohistochemical Staining

Paraffin-embedded tissue sections at 5 μm thickness were deparaffinized with xylene, dehydrated in gradually decreasing concentrations of ethanol, and then treated with 3% hydrogen peroxidase in methanol for 10 min to block endogenous peroxidase activity. Tissue sections were immersed in 10 mM sodium citrate buffer (pH 6.0) for 5 min at 95 °C. The last step was repeated using fresh 10 mM sodium citrate solution (pH 6.0). Sections were allowed to remain in the same solution while cooling for 20 min and rinsed in PBS. Sections were incubated with primary antibody (1:100 dilution) for 1 h at 37 °C. Primary antibodies were following: anti-TNF-α and anti-fibronectin (Abcam), anti-IL-1β (Santa Cruz), anti-TGF-β1 (R&D Systems). Signal was visualized using an Envision system (DAKO, CA, USA) for 30 min at 37 °C. DAB (3,3'-diaminobenzidine tetrahydrochloride) was used as the coloring reagent and hematoxylin was used as counter stain.

### 3.6. Immunofluorescent Staining

Paraffin-embedded tissue sections were deparaffinized with xylene and dehydrated in gradually decreasing concentrations of ethanol. Tissue sections were then placed in blocking serum (5% bovine serum albumin in PBS) at room temperature for 1 h. Primary antibody (1:500 dilution) was incubated at room temperature for 2 h, and secondary antibody incubation (1:200 dilution) was performed at room temperature for 2 h. Antibodies were following: α-SMA (Abcam), and goat anti-mouse IgG secondary antibody conjugated with FITC (Invitrogen, Carlsbad, CA, USA). Slides were mounted using VECTASHIELD Mounting Medium (VECTOR Laboratories, Burlingame, CA, USA). Specimens were examined and photographed using a fluorescence microscope (Nikon, Tokyo, Japan).

### 3.7. Reverse Transcription-Polymerase Chain Reaction (RT-PCR)

Total RNA was extracted from the frozen kidney with TRIzol Reagent (Gibco, Grand Island, NY, USA) according to the manufacturer’s recommendations. Purity and quantity of RNA preparation were measured at optical densities of 260 nm and 280 nm. First stand cDNA was synthesized with oligo-d(T) primer and M-MLV reverse transcriptase (Promega, Madison, WI, USA). Aliqout of cDNA was used for PCR using primer sets specific to mouse TNF-α, IL-1β, TGF-β1, fibronectin, and GAPDH. Primer sequences are following: TNF-α forward primer, 5'-AGT GGT GCC AGC CGA TGG GTT GT-3'; TNF-α backward primer, 5'-GCT GAG TTG GTC CCC CTT CTC CAG-3'; IL-1β forward primer, 5'-CAT GAG CAC CTT CTT TTC CT-3'; IL-1β backward primer, 5'-TGT ACC AGT TGG GGA ACT CT-3'; TGF-β1 forward primer, 5'-CCT GCT GCT TTC TCC CTC AAC C-3'; TGF-β1 backward primer 5'-CTG GCA CTG CTT CCC GAA TGT C-3'; fibronectin forward primer, 5'-TGT GAC AAC TGC CGT AGA CC-3'; fibronectin backward primer, 5'-GAC CAA CTG TCA CCA TTG AGG-3'; GAPDH forward primer, 5'-GTG GAC ATT GTT GCC ATC AAC G-3'; GAPDH backward primer, 5'-GAG GGA GTT GTC ATA TTT CTC G-3'. PCR products were visualized by 1.5% agarose gel electrophoresis with ethidium bromide staining.

### 3.8. Statistical Analysis

Data are presented as means ± SE. Student’s *t*-test was used to assess the significance of independent experiments. The criterion *p* < 0.05 was used to determine statistical significance.

## 4. Conclusions

This study demonstrated that natural toxin BV inhibits the development and progression of renal fibrosis in an animal model of UUO. Anti-fibrotic effects of BV are associated with inactivation of multiple cytokine and growth factors as well as inhibition of inflammatory responses. These results suggest that BV could have therapeutic potential for the treatment of renal fibrosis due to its interaction with multiple growth factor-mediated pro-fibrotic genes.
